# The “sweet” side of a long pentraxin: how glycosylation affects PTX3 functions in innate immunity and inflammation

**DOI:** 10.3389/fimmu.2012.00407

**Published:** 2013-01-07

**Authors:** Antonio Inforzato, Patrick C. Reading, Elisa Barbati, Barbara Bottazzi, Cecilia Garlanda, Alberto Mantovani

**Affiliations:** ^1^Department of Immunology and Inflammation, Humanitas Clinical and Research CenterRozzano, Italy; ^2^Department of Microbiology and Immunology, University of MelbourneMelbourne, VIC, Australia; ^3^Victorian Infectious Diseases Reference Laboratory, World Health Organization Collaborating Centre for Reference and Research on InfluenzaMelbourne, VIC, Australia; ^4^Department of Medical Biotechnology and Translational Medicine, University of MilanMilan, Italy

**Keywords:** pathogen recognition, inflammation, glycosylation, pentraxins, PTX3

## Abstract

Innate immunity represents the first line of defense against pathogens and plays key roles in activation and orientation of the adaptive immune response. The innate immune system comprises both a cellular and a humoral arm. Components of the humoral arm include soluble pattern recognition molecules (PRMs) that recognize pathogen-associated molecular patterns and initiate the immune response in coordination with the cellular arm, therefore acting as functional ancestors of antibodies. The long pentraxin PTX3 is a prototypic soluble PRM that is produced at sites of infection and inflammation by both somatic and immune cells. Gene targeting of this evolutionarily conserved protein has revealed a non-redundant role in resistance to selected pathogens. Moreover, PTX3 exerts important functions at the crossroad between innate immunity, inflammation, and female fertility. The human PTX3 protein contains a single N-glycosylation site that is fully occupied by complex type oligosaccharides, mainly fucosylated and sialylated biantennary glycans. Glycosylation has been implicated in a number of PTX3 activities, including neutralization of influenza viruses, modulation of the complement system, and attenuation of leukocyte recruitment. Therefore, this post translational modification might act as a fine tuner of PTX3 functions in native immunity and inflammation. Here we review the studies on PTX3, with emphasis on the glycan-dependent mechanisms underlying pathogen recognition and crosstalk with other components of the innate immune system.

## INTRODUCTION

The innate immune system has evolved to specifically recognize and eradicate potentially harmful microorganisms, thus providing the first line of defense against pathogens. Evolutionarily older than the adaptive immune system, innate immunity plays key roles in activation and orientation of adaptive immunity that provides the immunological memory. Components of the innate immune system that are involved in pathogen recognition and initiation of the immune response are germline-encoded receptors known as pattern recognition molecules (PRMs). These receptors recognize microbes or microbial moieties collectively named pathogen-associated molecular patterns (PAMPs; Iwasaki and Medzhitov, 2010). PRMs can also sense endogenous ligands termed “danger-associated molecular patterns (DAMPs),” which are released by host cells upon tissue damage during, for example, infection, inflammation, and tumor growth (Matzinger, 2002). As numerous PAMPs and DAMPs are glycans themselves or accommodate glycan structures, PRMs are mostly comprised of glycan-binding proteins or lectins. These include C-type lectins, siglecs, and galectins, which contain one or more carbohydrate-recognition domains (CRDs) that are responsible for sugar recognition (van Kooyk and Rabinovich, 2008). In addition, most PRMs are glycoproteins whose glycosidic moiety might have a dramatic impact on protein targeting and activity. For instance, some toll-like receptors (TLRs), such as TLR2 and TLR4, require glycosylation for correct cell compartmentalization (da Silva Correia and Ulevitch, 2002; Weber et al., 2004), and inhibition of glycosylation prevents TLR3-induced NF-κB activation, although it does not affect expression and trafficking of this receptor (Sun et al., 2006).

Considering cellular localization and function, PRMs are classified into two major groups: (i) cell-associated receptors, which are localized in different cellular compartments and include endocytic receptors, such as scavenger receptors (Murphy et al., 2005), signaling receptors, such as TLRs (Akira et al., 2006) and nucleotide-binding oligomerization domain (NOD)-like receptors (NLR; Meylan et al., 2006), and (ii) fluid phase molecules or opsonins, which represent the functional ancestors of antibodies and are involved in pathogen opsonization, complement activation, and self versus modified-self discrimination (Bottazzi et al., 2010). Fluid phase PRMs are essential effectors and modulators of the innate resistance in animals and humans, and form a heterogeneous group of molecules that includes collectins, ficolins, and pentraxins (Fujita, 2002; Holmskov et al., 2003; Bottazzi et al., 2006).

Conserved in evolution from arachnids to humans, pentraxins are pivotal components of the humoral arm of innate immunity with a distinctive multimeric structure. Based on the primary structure of the composing protomers, pentraxins are divided into two groups: short and long pentraxins (Deban et al., 2011). C-reactive protein (CRP) and serum amyloid P component (SAP) are prototypic short pentraxins, whereas pentraxin 3 (PTX3) and other subsequently identified proteins represent the long pentraxin arm of the family (Garlanda et al., 2005).

Here we review past and present literature on this family of proteins, with major emphasis on the long pentraxin PTX3 and the glycan-dependent mechanisms underlying its functions in pathogen recognition and crosstalk with other components of the innate immune system.

## THE SHORT PENTRAXINS

Pentraxins are distinctively characterized by the presence in their carboxy-terminal region of a ~200 amino acid domain containing a highly conserved motif of primary sequence known as pentraxin signature (HxCxS/TWxS, where x is any amino acid). In the 1930s, CRP was the first purified PRM, which was named after its ability to bind in a calcium-dependent fashion the C-polysaccharide of *Streptococcus pneumoniae *(Tillet and Francis, 1930; Abernethy and Avery, 1941). Human SAP was subsequently identified as a closely related protein (i.e., with 51% sequence identity to human CRP; Emsley et al., 1994). Orthologous proteins to human CRP and SAP have also been described in the hemolymph of the arthropod *Limulus polyphemus*, where they are involved in recognition and killing of pathogens (Liu et al., 1982; Armstrong et al., 1996; Shrive et al., 1999).

Both short pentraxins are 25 kDa proteins with a common structural organization that comprises 5 or 10 identical subunits arranged in a pentameric radial symmetry (Rubio et al., 1993; Emsley et al., 1994). Each of the composing subunits displays a lectin-like fold with two-layered β sheet in a flattened jellyroll topology (Thompson et al., 1999). The human CRP protein is not glycosylated, whereas human SAP is traditionally described as homogeneously modified with sialylated biantennary complex type glycans (Pepys et al., 1994). However, this view has been challenged by studies reporting microheterogeneity of the glycosidic moiety of SAP due to loss of one or both terminal sialic acid residues (Weiss et al., 2011). In this regard, it has been proposed that the loss of sialic acid might cause a reorientation of SAP oligosaccharides, which in turn induces conformational changes in the protein tertiary and quaternary structure. Therefore, it is conceivable that the glycosylation status of SAP might affect the structure/function relationships of this short pentraxin (Ashton et al., 1997; Siebert et al., 1997).

C-reactive protein and SAP are the main acute-phase reactants in human and mouse, respectively. CRP is barely detectable in the plasma of healthy human adults (í3 mg/l) but its concentration increases by as much as 1000-fold in several pathological conditions. As opposed to this, the concentration of human (but not murine) SAP is substantially invariant (30–50 mg/l), even during the early acute-phase response. Human CRP and SAP are both produced by hepatocytes, where the pro-inflammatory cytokine IL-6 is a major inducer of CRP both on its own and in synergy with IL-1 (Pepys and Hirschfield, 2003).

Short pentraxins are important players in humoral innate immunity, where they have been described to recognize a number of diverse ligands, mostly in a calcium-dependent manner. As mentioned above, the first reported ligand of CRP is the C-polysaccharide of *Streptococcus pneumoniae*. This interaction is mediated by phosphorylcholine (PC), a major constituent of the C-type capsule polysaccharides (Tillet and Francis, 1930; Abernethy and Avery, 1941). CRP recognizes additional pathogens, including fungi, yeasts, and bacteria, thus promoting phagocytosis and resistance to infection (Szalai, 2002). Consistent with this, CRP transgenic (Tg) mice are resistant to infection with *Streptococcus pneumoniae*, displaying longer survival time and lower mortality rate than normal littermates (Szalai et al., 1995). SAP is a calcium-dependent lectin originally purified based on its binding to the agarose component 4,6-cyclin pyruvate acetal of *β*-D-galactose (Hind et al., 1984). Like CRP, SAP binds a number of bacteria, such as *Streptococcus pyogenes *and *Neisseria meningitidis *(Hind et al., 1985; Noursadeghi et al., 2000). In addition it has been shown that SAP acts as β inhibitor against influenza virus, binding in a calcium-dependent fashion to mannose-rich glycans on the viral hemagglutinin (HA) to inhibit both hemagglutination and viral infectivity (Andersen et al., 1997; Horvath et al., 2001). Furthermore, SAP has been described to prevent lipopolysaccharide (LPS)-mediated complement activation and LPS toxicity (de Haas et al., 1998, 2000; Noursadeghi et al., 2000). However, the relationship between ligand binding and function of these proteins is still a matter of debate (Noursadeghi et al., 2000; Szalai et al., 2000; Suresh et al., 2007).

The short pentraxins participate in activation and regulation of the three complement pathways (i.e., classical, lectin, and alternative), by interacting with C1q (CRP and SAP; Roumenina et al., 2006), ficolins (CRP; Ng et al., 2007; Tanio et al., 2009), and factor H (CRP; Jarva et al., 1999; Mihlan et al., 2009; Okemefuna et al., 2010). It has been suggested that complement activation by short pentraxins might favor removal of the apoptotic debris, with potential implications in preventing the onset of autoimmune diseases (Nauta et al., 2003b). Specific and saturable binding to all three classes of Fcγ receptors (FcγR) has been demonstrated for both CRP and SAP, where these interactions mediate phagocytosis of apoptotic cells and microorganisms (Bharadwaj et al., 1999, 2001; Mold et al., 2002; Lu et al., 2008). Therefore, pentraxins can activate both complement and FcγR pathways, which resembles the functional properties of antibodies.

## THE LONG PENTRAXIN PTX3

Pentraxin 3 is the prototypic long pentraxin that was first identified in the early 1990s as a cytokine-inducible gene in endothelial cells and fibroblasts (Breviario et al., 1992; Lee et al., 1993). Long pentraxins have an unrelated amino-terminal region coupled to a 203 amino acids long carboxy-terminal pentraxin-like domain. Despite the sequence homology, long pentraxins differ from short pentraxins in gene organization, chromosomal localization, cellular source, inducing stimuli and recognized ligands. Other members of the long pentraxin subfamily have been identified, including guinea pig apexin (Reid and Blobel, 1994), neuronal pentraxin 1 (NPTX1 or NP1; Omeis et al., 1996), neuronal pentraxin 2 (NPTX2, also called Narp or NP2; Hsu and Perin, 1995), and the transmembrane protein neuronal pentraxin receptor (NPTXR; Dodds et al., 1997). In an attempt to find new pentraxin domain-containing proteins, we have recently identified a new long pentraxin, which we named PTX4. Like other members of this family, the gene encoding PTX4 is well conserved from mammals to lower vertebrates. However, PTX4 has a unique pattern of mRNA expression, which is distinct from that of other long pentraxins (Martinez de la Torre et al., 2010).

### GENE ORGANIZATION AND EXPRESSION

The human PTX3 gene has been localized on chromosome 3 band q25 and is organized in three exons, coding respectively for the leader peptide, the N-terminal domain and the pentraxin domain, separated by two introns. The murine gene presents the same structural organization and is located on chromosome 3. The proximal promoters of both human and murine PTX3 genes share numerous potential enhancer-binding elements, including Pu1, AP-1, NF-κB, SP1, and NF-IL-6 sites (Breviario et al., 1992; Altmeyer et al., 1995; Basile et al., 1997).******

Pentraxin 3 expression is rapidly induced in a variety of additional cell types by several stimuli, such as cytokines (e.g., IL-1β, and TNF-α), TLR agonists, microbial moieties (e.g., LPS, OmpA, and lipoarabinomannans), and intact microorganisms (Bottazzi et al., 2009). Oxidized and enzymatically modified low density lipoproteins (ox-LDL) promote PTX3 production in endothelial cells and human primary vascular smooth muscle cells (SMCs; Presta et al., 2007). Similar inflammatory signals induce PTX3 expression in other cell types, including myeloid dendritic cells (DCs), macrophages, kidney epithelial cells, synovial cells, chondrocytes, adipocytes, alveolar epithelial cells, glial cells, mesangial cells, and granulosa cells (Bottazzi et al., 2009). PTX3 is constitutively stored in the specific granules of neutrophils and is released in response to TLR engagement by microorganisms or TLR agonists (Jaillon et al., 2007; Maina et al., 2009). The released protein can partially localize in neutrophil extracellular traps (NETs) and neutrophil-associated PTX3 promotes the generation of an effective antimicrobial microenvironment (Jaillon et al., 2007). Constitutive expression of PTX3 has been reported in both human and murine lymphatic endothelial cells (Sironi et al., 2006), whereas resting T and B lymphocytes and natural killer cells do not produce PTX3 mRNA.

Different signaling pathways can affect PTX3 production, depending on cell type and/or stimuli. For instance, IFN-γ inhibits PTX3 production in DCs, monocytes, and macrophages, while IL-10 amplifies LPS-induced PTX3 expression (Doni et al., 2006; Maina et al., 2009). IL-4, dexamethasone, 1α, 25-dihydroxivitamin D3, and prostaglandin E2 also inhibit LPS-induced PTX3 in myeloid DCs (Doni et al., 2006). PTX3 expression in a model of acute myocardial ischemia is controlled by the NF-κB pathway (Salio et al., 2008), while induction of the protein by TNF-α in lung epithelial cells involves the c-Jun N-terminal kinase (JNK) pathway (Han et al., 2005). Moreover, production of PTX3 in endothelial cells that is induced by high-density lipoproteins (HDL) requires the activation of the PI3K/Akt pathway through G-coupled lysosphingolipid receptors (Norata et al., 2008). Glucocorticoid hormones (GCs) induce or enhance PTX3 production in non-hematopoietic cells (e.g., fibroblasts and endothelial cells), but inhibit PTX3 production in hematopoietic cells (e.g., DCs and macrophages; Doni et al., 2008).

### PROTEIN STRUCTURE AND GLYCOSYLATION

**FIGURE 1:**
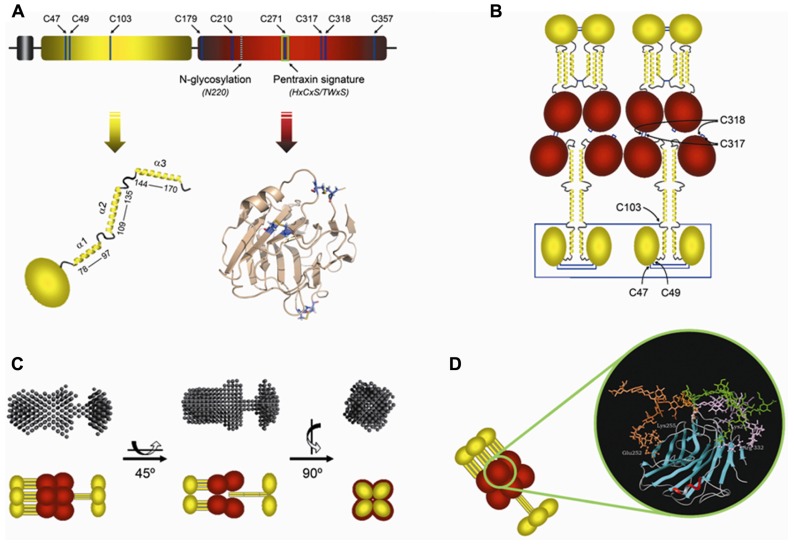
**Model of the PTX3 protein and its glycosylation**. **(A)** Schematic representation of the PTX3 protomer subunit showing the N-terminal domain in yellow, followed by the globular pentraxin domain in red. Positions of the Cys residues, the N-glycosylation site at Asn220 and the pentraxin signature motif are indicated. Arrows point to a topological drawing of the N-terminal domain, which is believed to be composed of a globular region and three α-helical segments (α1, α2, and α3), and a three-dimensional model of the C-terminal pentraxin domain based on the crystal structure of C-reactive protein (Protein Data Bank ID: 1b09). **(B)** Disulfide bond organization of the PTX3 octamer. Highlighted are the Cys residues that participate in disulfide bond formation. The α-helical regions of the N-terminal domains are predicted to form coiled-coil-like structures, which are hypothesized to adopt either an extended (bottom) or a compact (top) conformation. **(C)** Different views of the SAXS scattering envelope and a schematic model of PTX3 based on the two different structural arrangements proposed for the N-terminal domain. The α-helical segments of the N-terminal domain are depicted as yellow rods. The C-terminal pentraxin domains are in red. **(D)** Molecular dynamics simulations indicate that the PTX3 oligosaccharides, here represented by a core monofucosylated and desialylated biantennary glycan, can adopt different conformations (orange, green, and purple), where terminal residues of sialic acid can contact specific amino acids (ball-and-stick) at the protein surface (see text for details).

The human PTX3 is a multimeric glycoprotein whose composing subunits are made of 381 amino acids, including a 17-residue signal peptide (Breviario et al., 1992). PTX3 primary sequence is highly conserved among animal species (human and murine PTX3 sharing 92% of conserved amino acid residues), suggesting a strong evolutionary pressure to maintain its structure/function relationships. Like other members of the long-pentraxin family, PTX3 is composed of a unique N-terminal region and a 203 amino acid C-terminal domain homologous to the short pentraxins CRP and SAP (**Figure 1A**; Bottazzi et al., 1997).

The N-terminal region (residues 18–178 of the preprotein) is unrelated to any known protein structure. Nevertheless, secondary structure predictions indicate that this part of the protein is likely to form four α-helices, three of which (amino acids 78–97, 109–135, and 144–170) are probably involved in the formation of coiled-coil structures (Presta et al., 2007; Inforzato et al., 2010; **Figures 1A,B**). As stated above, the C-terminal domain of PTX3 (residues 179–381 of the preprotein) is homologous to the short-pentraxins CRP and SAP, with up to 57% similarity (Bottazzi et al., 1997). Therefore, three-dimensional models of this domain have been generated that are based on the crystallographic structures of CRP (PDB ID: 1b09) and SAP (PDB ID: 1sac; Goodman et al., 1996; Introna et al., 1996; Inforzato et al., 2006). Here we report a CRP-derived model (**Figure 1A**) that shows the pentraxin-like domain of PTX3 to adopt a β-jelly roll topology, similar to that found in legume lectins.

A single N-glycosylation site has been identified in the C-terminal domain of PTX3 at Asn220. This is fully occupied by complex type oligosaccharides, mainly fucosylated and sialylated biantennary sugars with a minor fraction of tri- and tetrantennary glycans. Based on three-dimensional models of the glycosylated C-terminal domain, we have proposed that the PTX3 oligosaccharides make contacts to polar and basic amino acids at the protein surface (i.e., Lys214, Glu252, Lys255, and Arg332) mainly through terminal residues of sialic acid (**Figure 1D**). These interactions are lost when sialic acid is removed and protein sites become accessible that are potentially relevant to ligands recognition and/or modifications of the PTX3 tertiary/quaternary structure (Inforzato et al., 2006). Interestingly, we have found that the relative content of bi-, tri-, and tetrantennary oligosaccharides and the level of sialylation are highly variable amongst PTX3 isolates from different cellular sources, thus suggesting that the glycosylation pattern of this long pentraxin might change depending on cell type and inducing stimuli. This might have important functional implications given that the glycosidic moiety of PTX3 has been involved in a number of biological activities as discussed below.

In addition to the multidomain organization, the human PTX3 protein shows a complex quaternary structure with eight protomer subunits assembled into high order oligomers stabilized by disulfide bonds (Inforzato et al., 2008; **Figure 1B**). Based on data from electron microscopy (EM) and small angle X-ray scattering (SAXS), we have generated a low-resolution model of the protein that shows the composing subunits to fold into an elongated structure with a large and a small domain interconnected by a stalk region (Inforzato et al., 2010; **Figure 1C**). This oligomeric state and the asymmetric shape of the molecule make PTX3 unique amongst pentraxins. Indeed, it displays pseudo 4-fold symmetry along its longitudinal axis, in sharp contrast to the typical pentameric arrangement of the classical short pentraxins. The structural determinants of the PTX3 quaternary organization are mainly localized in the protein N-terminal domain, where this region mediates the association of protomers into tetramers via both covalent (i.e., disulfide bonds) and non-covalent (i.e., inter-chain coiled-coils) interactions (see **Figure 1B**). Although a detailed discussion of the PTX3 structure is beyond the scopes of this review, evidence has been provided that the quaternary structure of this long pentraxin is fundamental in mediating its binding properties and, ultimately, its biological functions (Inforzato et al., 2011).

## PTX3 LIGANDS AND THE ROLE OF GLYCANS

The structural complexity and modular nature of PTX3, as described above, probably explain the rather broad spectrum of ligands of this long pentraxin and the diversity of its biological roles as compared to the short pentraxins (**Table 1**). Here we discuss the most relevant ligands of PTX3 based on the functional outcome of their interaction with this protein, with major emphasis on complement regulation, inflammation, and pathogen recognition. An effort has been made to revisit the PTX3 interactome in the light of the role that glycosylation plays in modulating and, in certain cases, dictating the protein functions.

**Table 1:** Ligand specificity of CRP, SAP, and PTX3.

Ligand	CRP	SAP	PTX3
**Complement components**			
C1q	+	+	+
Factor H	+	NT	+
C4BP	+	+	+
M-, L-ficolin	+	-	+
MBL	-	+	+
**Extracellular matrix proteins**			
TNF-stimulated gene-6 (TSG-6)	NT	NT	+
Inter-α-trypsin-inhibitor (IαI)	-	NT	+
Hyaluronan	NT	NT	-
Laminin	+	+	-
Collagen IV	NT	+	-
Fibronectin	+	+	-
**Growth factors**			
FGF2	+/-	NT	+
FGF1 and FGF4	NT	NT	-
**Membrane moieties**			
Phosphocholine (PC)	+	-	-
Phosphoethanolamine (PE)	-	+	-
LPS	-	+	-
Outer membrane protein A from *Klebsiella pneumoniae* (KpOmpA)	NT	NT	+
**Microorganisms**			
***Bacteria***			
*Pseudomonas aeruginosa*	NT	NT	+
*Klebsiella pneumoniae*	NT	NT	+
*Salmonella typhimurium*	-	+	+
***Fungi and yeasts***			
*Aspergillus fumigatus*	+	NT	+
*Saccharomyces cerevisiae* (Zymosan)	+	+	+
*Paracoccidioides brasiliensis*	NT	NT	+
***Viruses***			
Influenza virus	-	+	+
Human cytomegalovirus (HCMV)	NT	NT	+

*NT, not tested.*

### INTERACTION WITH COMPLEMENT

#### Classical pathway

The first described and best characterized ligand of PTX3 is the complement component C1q (Bottazzi et al., 1997; Nauta et al., 2003a). PTX3 binds to plastic-immobilized C1q (Nauta et al., 2003a; Roumenina et al., 2006), and, unlike the classical pentraxins, this interaction is calcium-independent and does not require previous aggregation of PTX3. Other components of the classical pathway of complement, such as C3 and C4, are not recognized by PTX3 (Bottazzi and Deban, unpublished observations). Interaction of C1q with plastic-immobilized PTX3, an experimental condition that mimics the surface of microbes, results in activation of the classical complement cascade, assessed as C3 and C4 deposition. On the other hand, the presence of PTX3 in solution causes a dose-dependent inhibition of the C1q hemolytic activity (Nauta et al., 2003a). These data indicate that PTX3 may exert a dual role and contrasting effects on complement activation: it supports clearance of material that is able to bind PTX3, such as microbes, while on the other hand it may protect against unwanted complement activation in the fluid phase (Inforzato et al., 2012).

The apparent ambivalence of PTX3 might have important implications in apoptosis, where rapid and efficient clearance of apoptotic cells by phagocytes is necessary to avoid tissue damage due to release of the pro-inflammatory content of dying cells (Jeannin et al., 2008). PTX3 and C1q bind apoptotic cells with similar kinetics, interacting with different binding sites and remaining stably associated to the apoptotic cell membrane (Baruah et al., 2006). The interaction of PTX3 with C1q in the fluid phase prevents C1q binding and C3 deposition onto apoptotic cells as well as the C1q-mediated phagocytosis of apoptotic cells by DCs and phagocytes (Rovere et al., 2000; van Rossum et al., 2004; Baruah et al., 2006). In contrast, when PTX3 is incubated with apoptotic cells, it enhances the deposition of both C1q and C3 on the cell surface (Nauta et al., 2003b). In addition, membrane-associated PTX3 acts as an “eat-me” molecule in promoting phagocytosis of late apoptotic neutrophils, as opposed to the soluble form of PTX3 that inhibits this process (Jaillon et al., 2009). This view is supported by data from an *in vivo* murine model of systemic lupus erythematosus, where it has been shown that PTX3 fosters the rapid clearance of apoptotic T cells by peritoneal macrophages (Lech et al., 2011).

**FIGURE 2:**
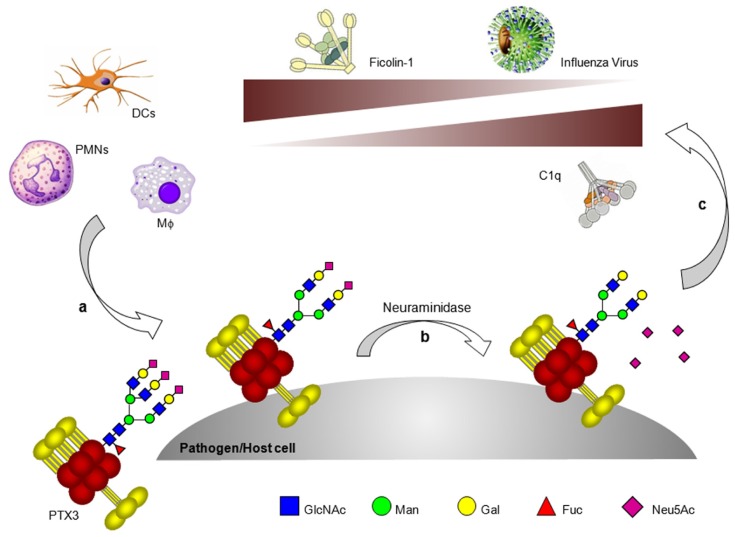
**Glycosylation as a tuner of PTX3 functions in innate immunity.** A number of both somatic and immune cell types produce PTX3 at sites of infection/inflammation. The glycosylation status of PTX3 (e.g., branching and sialylation) might change depending on cellular source and inducing stimuli **(a)**. In addition, the protein oligosaccharides might undergo processing by glycosidases, including neuraminidase, which are expressed or mobilized on the surface of both pathogens and host cells (e.g., neutrophils) **(b)**. Desialylated PTX3 has higher affinity for C1q but loses recognition of ficolin-1 and influenza virus **(c)**.

Interestingly, we have reported that the glycosylation status of PTX3 modulates the protein interaction with C1q mostly through the terminal residues of sialic acid. In fact, either desialylation or complete deglycosylation of the long pentraxin equally increase its binding to C1q (Inforzato et al., 2006). Consistent with this, hydrolysis of the terminal residues of sialic acid enhances the PTX3-dependent activation of the classical pathway of complement, as assessed by C3 and C4 deposition on PTX3-coated surfaces. Furthermore, in the fluid phase desialylated PTX3 is a stronger inhibitor of the C1q hemolytic activity than the fully glycosylated protein. Therefore the strengthening of PTX3 binding to C1q that occurs upon removal of sialic acid is independent of the way the long pentraxin is presented (i.e., either immobilized or in solution). Also, sialylation of the PTX3 oligosaccharides might provide a strategy to fine tune both the activating and inhibitory activities of this long pentraxin on the classical complement cascade (**Figure 2**).

#### Lectin pathway

We have recently found a direct interaction of PTX3 with ficolin-2, also known as L-ficolin (Ma et al., 2009), and ficolin-1, also named M-ficolin (Gout et al., 2011), where these molecules are major soluble receptors of the lectin pathway of complement. Both ficolins are ligands of CRP also, and a functional cooperation has been described with this short pentraxin that boosts complement-mediated antimicrobial activities (Ng et al., 2007; Zhang et al., 2009). Ficolin-1 and -2 bind PTX3 in a calcium-dependent fashion through their fibrinogen (FBG)-like domain. Ficolin-1 is a sialic acid-binding lectin; indeed enzymatic desialylation of PTX3 strongly impairs recognition of this long pentraxin by ficolin-1.

Also, mutants of this lectin with reduced binding to sialic acid display defective recognition of PTX3 (Gout et al., 2011). Ficolin-1 binds *Aspergillus fumigatus* and this interaction is strengthened by PTX3 and vice versa. Likewise, the ficolin-2-dependent deposition of complement components on the surface of *A. fumigatus* is enhanced by PTX3 (see below). This effect is alleviated by a common amino acid change in the FBG-like domain of ficolin-2, which affects recognition of *N*-acetyl-D-glucosamine (GlcNAc) and PTX3 (Ma et al., 2009). PTX3 and ficolin-2 may recruit each other to the surface of recognized microbes and amplify synergistically complement-mediated innate responses. PTX3 also triggers ficolin-1-dependent activation of the lectin complement pathway, although the potential physiological implications of this interaction remain to be determined (Gout et al., 2011). The crosstalk between PTX3 and ficolins is a remarkable example of how soluble PRMs can communicate through a common glycan code, where the functional information that is contained in the glycan structures of PTX3 is read and executed by the ficolins (**Figure 2**)

Recently an interaction of PTX3 with mannose-binding lectin (MBL), a molecule with structural and functional similarities with C1q and the ficolins, has been described (Ma et al., 2011). PTX3–MBL interaction occurs in a calcium-dependent manner and involves the MBL collagen-like domain. Formation of the MBL–PTX3 complex recruits C1q to *Candida albicans* and enhances C4 and C3 deposition as well as phagocytosis of this pathogen (Ma et al., 2011).

#### Complement regulation

In addition to components of the classical and lectin pathways of complement, PTX3 has been described to interact with factor H (FH; Deban et al., 2008), the main soluble regulator of the alternative pathway of complement activation, and with the classical and lectin pathway regulator C4b-binding protein (C4BP; Braunschweig and Jozsi, 2011). Two binding sites for PTX3 are present on FH: the primary binding site is located on FH short consensus repeat (SCR) domains 19–20, which interact with the N-terminal domain of PTX3, while a secondary site on SCR7 binds the glycosylated PTX3 pentraxin domain. In agreement, SCR7 has been also recognized as the CRP-binding site on FH (Jarva et al., 1999; Okemefuna et al., 2010). PTX3-bound factor H remains functionally active, and PTX3 enhances factor H and iC3b deposition on apoptotic cells. These observations suggest that the interaction of PTX3 with factor H modulates the alternative pathway activation by promoting factor H deposition on PTX3-coated surfaces and preventing exaggerated complement activation. The interaction with FH is strongly affected by PTX3 glycosylation, as indicated by the observation that deglycosylation of this long pentraxin impairs its binding to FH (Deban et al., 2008). It is therefore licit to speculate that the PTX3-dependent modulation of FH activity might be altered by the glycosylation status of the long pentraxin as discussed above for the C1q–PTX3 crosstalk.

Similarly to CRP and SAP, PTX3 binds C4BP (Braunschweig and Jozsi, 2011). This interaction is dependent on the presence of Ca^2+^ and inhibited by C1q and ficolin-2 but not by FH. Interaction of PTX3 with C4BP occurs without interfering with the cofactor activity of C4BP in the fluid phase. PTX3 can recruit C4BP on ECM and on apoptotic cells, where it can increase the rate of C4b-inactivation. In parallel, the deposition of the lytic C5b-9 terminal complex is reduced in the presence of PTX3 (Braunschweig and Jozsi, 2011). Thus PTX3 can recruit both complement regulators, FH and C4BP, to limit excessive complement activation on apoptotic cells.

Together, these observations suggest a dual role for PTX3 in the regulation of complement-mediated immune responses. Interplay with key components of the three complement pathways (i.e., C1q, ficolins, MBL, and factor H) points to PTX3 as an important player of the complex network of interactions that control complement functions. In addition, the functional outcome of these interactions is dependent on the glycosylation status of PTX3.

### INFLAMMATION

It has been shown that PTX3 plasma levels increase rapidly and dramatically in different pathological contexts. Overexpression of PTX3 confers to Tg mice greater resistance than wild-type animals to LPS toxicity and to cecal ligation and puncture (Dias et al., 2001). PTX3 can also modulate inflammation in sterile conditions. In agreement, PTX3 deficiency is associated to more severe tissue damage in experimental models of kainate-induced seizures (Ravizza et al., 2001), cardiac ischemia and reperfusion (Salio et al., 2008), pleurisy and acute lung and kidney injury (Deban et al., 2010; Han et al., 2012; Lech and Garlanda, unpublished observation). Lack of PTX3 promotes autoimmune lung damage in *Fas*-deficient (lpr) mice (Lech et al., 2011), and accumulation of macrophages, neutrophils and CD3^+^ cells. Finally, deficiency of PTX3 on an apolipoprotein E knock-out background is associated with increased atherosclerosis, macrophage accumulation within the plaque, and a more pronounced inflammatory profile in the vascular wall (Norata et al., 2009). Higher macrophage and neutrophil accumulation has been also observed after coronary artery ligation and reperfusion in *ptx3*^-/-^ mice, associated to greater myocardial lesion, higher no-reflow area, and more apoptotic cardiomyocytes (Salio et al., 2008). In contrast with these results, PTX3 is deleterious in a model of ischemia and reperfusion of the superior mesenteric artery, increasing tissue damage and mortality (Souza et al., 2002, 2009), and accelerates lung injury in high tidal volume ventilation in mice (Real et al., 2012). These data indicate that PTX3 has a critical impact as fine tuner of inflammation, with both detrimental and beneficial effects, depending on the nature of the insult.

Different mechanisms may be involved in the regulatory functions of PTX3 in inflammatory reactions. PTX3 binds to dying neurons, rescuing them from irreversible damage and thus conferring resistance to neurodegeneration. The higher resistance to LPS observed in *Ptx3*-Tg mice has been attributed, at least in part, to higher IL-10 and NO production by peritoneal macrophages while a stronger deposition of complement component C3 was observed in the infarct area of *ptx3*^-/-^ mice, suggesting that the modulation of the complement cascade likely contributes to the cardioprotective role of PTX3 (Salio et al., 2008).

The protective role exerted by PTX3 in the lung injury and pleurisy models is selectively linked to the capacity of PTX3 to bind P-selectin, thus inhibiting leukocyte rolling on endothelium. The N-linked glycosidic moiety of PTX3 is essential for the interaction with P-selectin (Deban et al., 2010). In fact enzymatic deglycosylation of PTX3 or site-directed mutagenesis of Asn220 (i.e., the N-linked glycosylation site) resulted in 70% reduction of PTX3 binding to P-selectin. In addition lack of PTX3 glycosylation reversed the inhibitory effect of the wild-type molecule in the pleurisy model. Therefore, the regulatory function of PTX3 on inflammation is mediated by the protein glycosidic moiety, which is reminiscent of some properties of the immunoglobulins (Kaneko et al., 2006).

As mentioned above, PTX3 has an ambivalent role in the apoptotic process *in vitro. In vivo*, *ptx3*^-/-^ mice have a defective capability to clear apoptotic T cells (Lech et al., 2011). In addition lack of PTX3 on lupus prone genetic background (B6lpr) accelerates the evolution of autoimmune lung disease, which is associated to selective expansion of CD4/CD8 double negative “autoreactive” T cells (Lech et al., 2011). Thus PTX3 may be also involved in resolution of inflammation through the complex role exerted on the apoptotic process, this function being relevant for the control of autoimmunity.

### PATHOGEN RECOGNITION

Pentraxin 3 interacts with selected microbes, including fungi like *Saccharomyces cerevisiae*, *Paracoccidioides brasiliensis* and conidia from* A. fumigatus*, bacteria as *Pseudomonas aeruginosa* and *Streptococcus pneumoniae*, and viruses, such as human and murine cytomegalovirus (HCMV and MCMV) and the H_3_N_2_ influenza virus (Garlanda et al., 2002; Diniz et al., 2004; Gaziano et al., 2004; Bozza et al., 2006; Reading et al., 2008; Moalli et al., 2011a). Interestingly, the binding of PTX3 to *A. fumigatus *conidia is abolished in the presence of galactomannan, a major constituent of the conidium wall, but not in the presence of dextran, galactose, fucose, or mannose (Garlanda et al., 2002). However, a direct interaction between PTX3 and galactomannan has not been proved as yet.

*In vivo* studies with *Ptx3*-deficient mice showed that PTX3 plays non-redundant roles in innate resistance to infections caused by these microorganisms, and for some of them the protective role of PTX3 is mediated by complement. *Ptx3*-deficient mice are highly susceptible to invasive pulmonary aspergillosis, showing high mortality (Garlanda et al., 2002). *Ptx3*-deficient phagocytes exhibit defective recognition and killing of conidia and the treatment with recombinant PTX3 or neutrophil-associated PTX3 reverses this phenotype *in vitro* and *in vivo* (Garlanda et al., 2002; Gaziano et al., 2004; Jaillon et al., 2007; D’Angelo et al., 2009). The molecular mechanisms underlying the opsonic activity of PTX3 and increased phagocytosis of conidia by neutrophils involve Fcγ receptor II (FcγRII)-, CD11b-, and complement-dependent mechanisms (Moalli et al., 2010). Indeed, PTX3 modulates different effector pathways involved in innate resistance to *A. fumigatus*, including complement activation (Bottazzi et al., 1997; Nauta et al., 2003b; Deban et al., 2008; Ma et al., 2009) or promotion of phagocytosis by interacting with FcγRs, which have been identified as pentraxin receptors (Lu et al., 2008).

Recent results indicate that PTX3 has therapeutic activity in chronic lung infections by *Pseudomonas aeruginosa*, a major cause of morbidity and mortality in cystic fibrosis (CF) patients. Treatment with recombinant human PTX3 causes enhanced clearance of bacteria from the lungs of chronically infected mice, reduced production of local pro-inflammatory cytokines and chemokines, neutrophil recruitment in the airways and histopathological lesions. Also in this condition, the PTX3-dependent recognition and phagocytosis of *Pseudomonas aeruginosa* involves the interplay with complement and FcγRs (Moalli et al., 2011b).

Pentraxin 3 binds to the outer membrane protein A (KpOmpA), a major component of the outer membrane of *Klebsiella pneumoniae* (Jeannin et al., 2005), which interacts also with different cellular PRMs, such as scavenger receptor lectin-like oxidized low-density lipoprotein receptor-1 (LOX-1) and scavenger receptor expressed by endothelial cell-I (SREC-I), and TLR2. The recognition of this microbial component by PTX3 amplified the inflammatory response through complement. Thus, recognition and activation of several PRMs by KpOmpA lead to amplification of the cellular and humoral innate responses to this microbial moiety (Jeannin et al., 2005; Cotena et al., 2007).

Bozza et al. (2006) studied the role of PTX3 in viral infections and found that PTX3 binds both HCMV and MCMV, reducing viral entry and infectivity in DC *in vitro*. Consistent with this *Ptx3*-deficient mice are more susceptible to MCMV infection than wild-type mice and PTX3 protects susceptible BALB/c mice from MCMV primary infection and reactivation *in vivo* as well as *Aspergillus *superinfection (Bozza et al., 2006). Recent studies also demonstrated that PTX3 binds to coronaviruses such as murine hepatitis virus (MHV)-1/3 and reduced the infectivity of MHV-1 *in vitro*. Moreover, *Ptx3*-deficient mice showed enhanced susceptibility to MHV-1 pulmonary infection and administration of exogenous PTX3 accelerated viral clearance, reduced neutrophil influx and ameliorated lung injury (Han et al., 2012).

Perhaps the best characterized viral ligand of PTX3 is influenza A virus (IAV) (Reading et al., 2008). Given the major focus of this review and the prevalent role of PTX3 glycosylation in the interaction with IAV, this topic will be discussed in a separate chapter (see below).

The relevance of the role of PTX3 in infections in humans has been demonstrated by Olesen et al. (2007), who recently showed that polymorphisms within the PTX3 gene and in particular the frequency of specific PTX3 haplotypes were significantly different in pulmonary tuberculosis patients as compared to healthy individuals. Along the same line, Chiarini et al. (2010) showed that the polymorphisms studied in tuberculosis correlated also with the risk of *Pseudomonas aeruginosa* infections in CF patients. PTX3 haplotype frequencies differed between CF patients colonized or not by *Pseudomonas aeruginosa*, and a specific haplotype was associated with a protective effect.

## PTX3 AND INFLUENZA VIRUS

Pentraxin 3 acts as a sialylated glycoprotein inhibitor of IAVs both *in vitro* and *in vivo *(Reading et al., 2008). The sialylated glycan of PTX3 is recognized by the HA of susceptible IAV strains resulting in inhibition of virus-induced hemagglutination and neutralization of virus infectivity (**Figure 2**). In this way PTX3 acts as a γ-type inhibitor, providing ligands that mimic the structure of the cellular receptors used by IAV to attach to the surface of target cells. PTX3 also interacts with HA expressed on the surface of IAV-infected cells and could therefore act as a direct opsonin, or promote deposition of complement components, to facilitate uptake and destruction by phagocytic cells expressing FcγRs or complement receptors, respectively. Of interest, binding of the viral HA to PTX3 also blocks the enzyme function of the viral neuraminidase (NA), the other major surface glycoprotein of IAV. By inhibiting NA-mediated cleavage of sialic acids, PTX3 may inhibit release of newly-formed virions from the surface of infected cells in a manner analogous to that of NA-specific antibodies (Kilbourne et al., 1968) or NA inhibitor drugs (Gubareva et al., 2000). Although enzymatic removal of sialic acid from PTX3 potentiates its ability to activate the classical complement pathways (Inforzato et al., 2006), desialylated PTX3 is not recognized by IAV and its anti-IAV activity is lost (Reading et al., 2008).

Human ficolins-1/2/3 bind IAV to neutralize virus infectivity (Pan et al., 2012; Verma et al., 2012) and the antiviral activities of ficolins-1/3 are augmented in the presence of PTX3 (Verma et al., 2012). PTX3 may also potentiate the anti-IAV activity of other innate immune proteins. For example, MBL mediates potent anti-IAV activity, including complement-mediated neutralization and lysis of IAV-infected cells (Anders et al., 1994; Reading et al., 1995) and complexes comprising MBL and PTX3 recruit C1q to amplify complement activation via the classical pathway (Ma et al., 2011). Conceivably, MBL–PTX3 hetero-complexes bound to IAV virions and/or IAV-infected cells may also augment complement activation to promote opsonization and/or lysis.

Attachment of IAV to the surface of target cells is mediated by the viral HA, which recognizes terminal sialic acid residues expressed by cell-surface glycoproteins and glycolipids. In general terms, the HA of avian IAV prefers Neu5Acα2-3Gal-containing receptors, which are expressed throughout the digestive and respiratory tracts of birds. In contrast, human IAV bind receptors containing Neu5Acα2-6Gal, the predominant linkage expressed by epithelial cells of the human upper airways (reviewed in Wilks et al., 2012). In the 20th century, three subtypes of human IAV (H1N1, H2N2, and H3N2) evolved from pandemic viruses of 1918, 1957, and 1968, respectively, and all originated from avian viruses that transmitted to humans. Soon after their introduction into humans in 1968, H3N2 IAV displayed dual HA specificity for Neu5Acα2-3Gal and Neu5Acα2-6Gal (Ryan-Poirier et al., 1998), but from 1975 onward isolates recognized only Neu5Acα2-6Gal. Early H3N2 strains (1968–1973) were sensitive to PTX3 whereas later strains (>1975) were resistant (Reading et al., 2008), consistent with exclusive expression of Neu5Acα2-3Gal by recombinant PTX3. To date, the antiviral activity of PTX3 against avian IAV has not been reported. However, given the HA preference of avian strains for Neu5Acα2-3Gal, the neutralizing activity of PTX3 in human airways may represent an additional factor limiting interspecies transmission. Moreover, in the first years of a pandemic when receptor specificity can be less strict (Ryan-Poirier et al., 1998; Matrosovich et al., 2000), PTX3 may be one factor driving evolution of human IAV with dual specificity toward preferential recognition of Neu5Acα2-6Gal.

Given the HA-mediated receptor specificity, the PTX3 glycosylation will be critical in determining its spectrum of anti-IAV activities. Moreover, glycans terminating with sialic acid represent attachment factors and/or receptor components for many different viruses (reviewed in Neu et al., 2011), suggesting that the γ-type antiviral activity of PTX3 may not be limited to IAV. Recombinant PTX3 expressing Neu5Acα2-3Gal mediates potent anti-IAV activity against certain H3N2, but not H1N1, virus strains *in vitro* (Reading et al., 2008; Job et al., 2010). In addition to the specificity of HA, the viral NA plays a critical role in determining PTX3 sensitivity, as the sialic acid moiety on PTX3 must resist hydrolysis by NA for neutralization to occur. Therefore, specificity and activity of N1 versus N2 NA might be an additional factor contributing to the differences observed in the sensitivity of H1N1 and H3N2 viruses to PTX3. PTX3 preparations from human DCs and fibroblasts show heterogeneity in the relative amounts of bi-, tri-, and tetrantennary glycans (Inforzato et al., 2006). Therefore, it will be important to define the sialic acid content and linkage of natural PTX3 from additional cellular sources (e.g., from neutrophils) and how this modulates the anti-IAV activity of PTX3.

Pentraxin 3 is highly conserved in evolution and murine and human PTX3 are equally effective in mediating anti-IAV activity (Reading et al., 2008). PTX3 production is upregulated in mice following IAV infection and *Ptx3*-deficient mice are more susceptible to a PTX3-sensitive H3N2 strain demonstrating the importance of endogenous PTX3 in the murine model of IAV infection. Moreover, treatment of wild-type mice with recombinant human PTX3 ameliorates virus replication and disease severity in mice infected with PTX3-sensitive virus, providing the first evidence of the therapeutic potential of PTX3 against IAV infections *in vivo*. In addition to binding IAV and facilitating virus clearance, PTX3 may also regulate inflammatory responses to IAV infection in the lung and further studies are required to define the mechanisms by which endogenous and exogenous PTX3 ameliorate disease in the mouse model of IAV infection.

## CONCLUDING REMARKS

Pentraxin 3 is an evolutionary conserved element of the humoral arm of the innate immune system, which plays a non-redundant role in resistance against selected pathogens, such as *A. fumigatus* and influenza virus (Garlanda et al., 2002; Reading et al., 2008). It binds microbial moieties (Jeannin et al., 2005), orchestrates complement activation (Doni et al., 2012), and has opsonic activity (Moalli et al., 2010). The single N-linked glycosylation site present in the molecule is occupied by complex type sialylated oligosaccharides that have been shown to modulate PTX3 crosstalk with a number of its ligands (i.e., complement components, influenza virus, and P-selectin).

As a consequence, these data point to a critical role of glycosylation in the modulation of PTX3 functions. It has been suggested that PTX3 can act as double edged sword. Following binding to microbial moieties, it has opsonic activity, activates complement and enhances leukocyte recruitment. On the other hand, PTX3 *per se* can act as a regulatory molecule of innate immunity and inflammation by dampening excessive neutrophil recruitment. This latter activity in particular is clearly dependent on PTX3 glycosylation (Deban et al., 2010).

The finding of a regulatory function mediated by the glycosidic moiety is reminiscent of similar activity of immunoglobulins (Kaneko et al., 2006). Thus, molecules of the humoral arm of the innate immune system, such as PTX3, and immunoglobulins, share fundamental mechanisms of activity, including agglutination, complement activation, opsonization, and glycosylation-dependent regulation of inflammation, despite the genetic and structural differences between the two classes of molecules.

## Conflict of Interest Statement

The authors declare that the research was conducted in the absence of any commercial or financial relationships that could be construed as a potential conflict of interest
